# Postpartum Depressive Symptoms: An Analysis of Social Determinants Using the Pregnancy Risk Assessment Monitoring System

**DOI:** 10.1089/whr.2023.0050

**Published:** 2023-12-04

**Authors:** Sarah O'Connor, Lihchyun Joseph Su

**Affiliations:** Peter O'Donnell Jr. School of Public Health, UT Southwestern Medical Center, Dallas, Texas, USA.

**Keywords:** postpartum depression, postpartum depressive symptoms, PRAMS, social determinants of health

## Abstract

**Background:**

Approximately one in every eight mothers experience symptoms of postpartum depression (PPD) in the United States.^1^ Existing literature lacks an in-depth exploration of the social context from which symptoms of PPD arise. The objectives of this study were to (1) determine the prevalence of postpartum depressive symptoms (PDS) among new mothers and to explore relationships between selected social determinants of health (SDOH) and the likelihood of experiencing PDS.

**Materials and Methods:**

Data were from the Pregnancy Risk Assessment Monitoring System (PRAMS) 2016 and 2017 Questionnaires. Measured SDOH included socioeconomic status, social network support, psychosocial stress, and availability of resources to meet basic daily needs. Outcome measurement included a combination of two symptom indicator questions. Univariate analyses yielded weighted frequencies of descriptive statistics according to PDS status, and bivariate and multivariate logistic regression analyses yielded odds of reporting PDS.

**Results:**

The prevalence of self-reported PDS was 3.5%. Among mothers with PDS, most (54%) lived at or below the federal poverty guideline. Mothers who experienced psychosocial stress (*e.g.*, intimate partner violence) during pregnancy had the highest likelihood of reporting PDS (adjusted odds ratio [aOR] = 3.60; confidence interval [95% CI], 2.12–6.12). Mothers who considered their most recent pregnancy unintended or mistimed were more likely to report PDS (aOR = 1.36; 95% CI, 1.01–1.82), (aOR = 1.65; 95% CI, 1.19–2.27), respectively.

**Conclusion:**

Results demonstrate that several social and psychosocial risk factors significantly impact the likelihood of experiencing PDS. The risk of PDS was particularly significant among lower socioeconomic status mothers, especially those with inadequate social network support. Public health efforts to mitigate potentially harmful social factors should focus on transforming public policies and social programs and increasing screening opportunities.

## Introduction

Postpartum depression (PPD) is a dangerously prevalent condition that affects approximately one in every eight new mothers in the United States.^1^ That number can be as high as one in five, depending on the state in which the mother lives.^1^ Given its burden and potentially long-term implications for mothers and their infants, it is critical for research to not only identify individual-level risk factors for experiencing depressive symptoms during the postpartum period but also to address the societal contexts from which these symptoms derive.

Following childbirth, levels of estrogen and progesterone in a mother's body decrease rapidly.^2^ This abrupt hormonal shift leads to changes in her brain that may trigger fluctuations in mood, and such changes are expected. However, persistent feelings of extreme sadness, loss of interest in usually enjoyable activities, or difficulty bonding with the baby indicates PPD.^2^

Ever-increasing evidence implies that the postpartum period has substantial and long-lasting implications for mothers, their children, and their families.^3,4^ Mothers with PPD are less likely to initiate and sustain breastfeeding and use safe sleep practices.^5,6^ Depression adversely impacts the mother–infant dyad by limiting the mother's ability to respond emotionally and bond with the infant.^7^ The quality of the mother–infant dyad plays a critical role in the infant's social, cognitive, and behavioral development.^4,7,8^

Psychological risk factors such as antenatal depression (*i.e.*, depression during pregnancy) have been well-established as a powerful predictor of PPD.^9,10^ In addition to biological risk factors, previous research has demonstrated that experiencing stressful life events (*e.g.*, financial hardship and intimate partner violence [IPV]) before and during pregnancy increase the risk of developing PPD,^11–14^ the existing literature lacks an in-depth exploration of the social context from which such stressful life events might arise. Examining the associations between social determinants of health (SDOH) and postpartum depressive symptoms (PDS) is critical to developing supportive public health policies and social programs to reduce the burdensome symptoms of PPD among new mothers.

According to the U.S. Department of Health and Human Services, SDOH are the conditions in the environments where people are born, live, learn, work, play, worship, and age that affect a wide range of health, functioning, and quality-of-life outcomes and risks.^15^ SDOH can be grouped into five domains: economic stability, education access and quality, health care access and quality, neighborhood and built environment, and social and community context.^15^ SDOH significantly impact people's health, well-being, and quality of life. Examples of SDOH include safe housing, transportation, racism, violence, education, job opportunities, income, and social cohesion. Recent literature has demonstrated that maternal health outcomes are markedly worse when pregnant and postpartum women are housing insecure, hungry, face financial instability, lack workplace protections and benefits, or are repeatedly exposed to crime and violence, including sexual assault and intimate partner violence.^16^

For this study, the social context surrounding each mother was measured by her socioeconomic status (*e.g.*, education, income, and occupation), social network (*e.g.*, marital status, IPV), psychosocial stress (*e.g.*, pregnancy intendedness, IPV), and availability of resources to meet basic daily needs (*e.g.*, prenatal care health insurance, Women, Infants, and Children [WIC] eligibility).

## Materials and Methods

### Study design and participants

This secondary cross-sectional study analyzed data collected in utilized data from the 2016 and 2017 Pregnancy Risk Assessment Monitoring System (PRAMS). PRAMS is an ongoing state-based surveillance system conducted annually by the Centers for Disease Control and Prevention's (CDC) Division of Reproductive Health in collaboration with state health departments. PRAMS is designed to identify and monitor maternal behaviors, emotions, and experiences before, during, and following a live birth. PRAMS uses a complex survey design to capture adequate information from vulnerable populations.^17^ Participating states utilize birth certificate records as the sampling frame for identifying a representative sample of between 100 and 250 new mothers each month. Mothers' survey responses are linked to extracted birth certificate variables, yielding data that contain a wealth of demographic information.

Currently, PRAMS surveillance represents ∼83% of all live births in the United States.^17^ All women who delivered a live infant during the surveillance period (2016 and 2017) and reported their most recently born infant as alive at the time of survey completion were eligible for inclusion (*N* = 64,421).

### Measures

Data were from 30 participating states^*^, which met the response threshold requirement while implementing the PRAMS Phase 8 Core Questionnaire.

#### Postpartum depressive symptoms

PPD may begin at any time after childbirth but commonly begins between 1 and 4 weeks after delivery.^2,3^ PRAMS participants are sampled and complete the questionnaire between 2 and 6 months after birth. Knowing that the expected changes in a new mother's emotional state are likely to be resolved within a few weeks postpartum, any depressive symptoms that have persisted until the time of questionnaire completion are highly suggestive of PPD. The PRAMS questionnaire includes core questions implemented by all participating states and state-specific questions chosen or developed by individual states to supplement the core questionnaire. From 2016 to the present, the core questionnaire has included two questions relating to symptoms of PPD.

The outcome of interest was defined by participants' responses to both the symptom indicator questions related to self-assessed experience with symptoms of PPD. Mothers were asked, “Since your new baby was born, how often have you felt down, depressed, or hopeless?” to which they were asked to rate the frequency of experiencing the feeling as always, almost always, or often, sometimes, rarely, or never. Next, mothers were asked, “How often have you had little interest or little pleasure in doing things you usually enjoyed?” to which they were asked to rate the frequency with the same response choices as the previous indicator question. To remain consistent with the methodology of existing literature,^1^ mothers indicating they “always” or “almost always” experienced both symptoms were classified as experiencing PDS; those reporting that they sometimes, rarely, or never experienced both symptoms were classified as not experiencing PDS.

#### Social determinants of health

Determining which and how to measure SDOH was complex and cumbersome. Each of the five SDOH domains includes many related variables, and each has varying degrees of influence on maternal mental health outcomes. In addition to young age and low socioeconomic status, single marital status (*i.e.*, unwed or separated/divorced) and low educational attainment have increased women's risk for PPD.^18–20^ A contributor to the association between single marital status and PPD could be a lack of sufficient father involvement during pregnancy and in parenting after birth.^21,22^ For this study, predictor or SDOH variables were limited to the scope of the questionnaire. Every attempt was made to measure SDOH similarly to previous studies for consistency and reproducibility.

Thus, the social context surrounding each mother was measured by her socioeconomic status (*e.g.*, education, income, and occupation), social network (*e.g.*, marital status, IPV), psychosocial stress (*e.g.*, pregnancy intendedness, IPV), and availability of resources to meet basic daily needs (*e.g.*, prenatal care health insurance, WIC eligibility).

#### Socioeconomic status

Socioeconomic status is typically characterized along the following three dimensions: education, income, and occupation.^23^ Maternal education was classified as less than high school, high school graduate/GED, and greater than high school. Income was a self-reported value that referred to total household income before taxes in the 12 months that preceded delivery. Income ranges were created for classification purposes and to distinguish mothers living at or below the Federal Poverty Guidelines (FPG) established by the U.S. Department of Health and Human Services. For a family of 2 in 2016 and 2017, 100% of the FPG was $16,020 and $16,240, respectively.^24^ Furthermore, 138% of the FPG (considered eligible for Medicaid) for a family of 2 in 2016 and 2017 was $22,108 and $22,411, respectively.^24^

While the scope of the questionnaire allowed for simple assessments of education and income, measuring occupation was more challenging. *In lieu* of employment status data, occupation and occupational network support during the postpartum period were measured by breastfeeding and pumping status. Adherence to breastfeeding is contingent mainly on the quantity and quality of maternal occupational leave time and an accommodating occupational environment with a suitable location and policies that allow time for pumping. Work-related issues have been cited as a major reason for noninitiation^25–31^ and early cessation of breastfeeding.^25–27,32^

#### Social network

Measuring the mother's social network was challenging due to the limitations of the questionnaire. Thus, marital status was a proxy for spousal support during the postpartum period. Participants reported being married or unmarried. Single marital status (*i.e.*, unwed or separated/divorced) has been found to increase a woman's risk for PPD.^18–20^ A contributor to the association between single marital status and PPD could be a lack of sufficient father involvement during pregnancy and in parenting after birth.^21,22^ The quantity of previous full-term pregnancies (*i.e.*, parity) indicated how many dependents each mother had in her social network before her most recently delivered. Previous research has demonstrated that women with multiparity had a higher risk of PPD, especially if their social support was low.^33–35^ It is plausible that multiparity could increase maternal stress and depressive symptoms because of multiple dependents' increased physical, emotional, and financial demands.

Whether or not the participant experienced IPV during her most recent pregnancy served as a measure of the quality of her social network and an indicator of psychosocial stress. Intimate partner violence during pregnancy presents a risk for maternal mental health problems, preterm birth, and having a low birth weight infant.^36^ A 2012 meta-analysis by Beydoun et al. demonstrated a 1.5–2-fold increased risk of elevated PDS and PPD among women exposed to IPV relative to nonexposed women.^37^

#### Availability of resources to meet daily needs

Insurance status can affect whether a pregnant woman seeks medical care and how often, the services and facilities available to her, and ultimately her health and that of her infant.^38^ Both cross-sectional studies and natural experiments provide evidence of the influence of insurance status on prenatal care.^38^ At the time of prenatal care, health insurance type was reported as private, Medicaid, self-pay, or no prenatal care. Pregnancy intendedness served as an additional measure of psychosocial stress and socioeconomic status. It has been estimated that nearly half of all pregnancies in the United States are unintended, and poorer women are disproportionately affected.^39,40^ Limited access to family planning services (*e.g.*, condoms, pharmaceutical contraceptives) increases rates of unintended pregnancies.^39^ Pregnancy intention was classified as intended, mistimed, and unintended.^39^

Recent literature has indicated that WIC participants are at significantly higher risk for PDS than ineligible women.^41^ This is likely due to WIC participation being a proxy for household income and socioeconomic status. Participants indicated whether they were eligible for WIC supplemental nutritional assistance during pregnancy.

### Statistical analyses

Survey procedures with SAS version 9.4 (Cary, NC) were utilized to account for the complex survey weights while conducting statistical analyses. PRAMS data were weighted to ensure that the sample represented all women who had a live birth in the United States between 2016 and 2017. Participants were excluded from analyses if data were missing for one or both symptom indicator questions. Descriptive statistics were used to summarize weighted frequencies of baseline sociodemographic characteristics by PDS status. Using bivariate regression models, unadjusted odds ratios (ORs) were calculated to predict the odds of experiencing PDS. Mothers with a history of depression during pregnancy were significantly more likely to report PDS. Thus, for a final subanalysis, mothers who experienced PDS were stratified by antenatal depression status.

Using multivariate logistic regression models to control for the confounding effects of maternal age, race, and ethnicity, adjusted ORs (aORs) were calculated to predict the odds of experiencing PDS among mothers with different socioeconomic circumstances.

## Results

Among the 66,232 mothers who completed the PRAMS Phase 8 Core Questionnaire, 64,421 completed both the symptom indicator questions and were included in the analysis. The prevalence of self-reported PDS was 3.5%. The highest state-specific prevalence of PDS (6.8%) was among Arkansan mothers, a notable 3.3% higher than the average. Mothers in New York City reported the lowest prevalence (1.5%) of PDS.

Among mothers who reported PDS, most were unmarried (54.3%), 20 to 29 years old (58.8%), not currently breastfeeding (57.6%), had annual household incomes of less than $24,000 (51.2%), and utilized Medicaid as their source of health insurance to access prenatal care (52.9%) (Table 1). Among mothers who did not report PDS, most were married (63.2%), 20 to 29 years old (47.5), currently breastfeeding (60.2%), had annual household incomes of greater than $60,000 (34.2%), and utilized a private source of health insurance to access prenatal care (44.0%) (Table 1). Approximately 62% of mothers experiencing PDS reported having a clinical visit for depression during their most recent pregnancy (Table 1).

**Table 1.** Sociodemographic Characteristics of Pregnancy Risk Assessment Monitoring System Participants by Postpartum Depressive Symptoms, 2016 to 2017 (*N* = 64,421)

Characteristics^a^	PDS (***n*** = 2,242)	No PDS (***n*** = 62,179)
	***n* (weighted %)**	***n* (weighted %)**
Maternal age, years		
19 and younger	151 (6.4)	3,076 (4.7)
20–29	1,260 (58.8)	29,835 (47.5)
30–39	779 (33.0)	27,177 (44.5)
40 and older	52 (1.8)	2,090 (3.3)
Race		
White	1,367 (72.6)	36,872 (70.2)
Black	456 (15.2)	11,512 (14.4)
Other^b^	398 (12.2)	13,101 (15.4)
Ethnicity		
Non-Hispanic	1,916 (84.7)	49,814 (80.0)
Hispanic	322 (15.3)	12,007 (20.0)
Education		
Less than high school	305 (12.3)	8,196 (12.7)
High school graduate/GED	701 (32.0)	14,802 (23.8)
Greater than high school	1,215 (55.7)	38,550 (63.5)
Annual household income^c^		
$0–$16,000	709 (32.6)	11,681 (20.9)
$16,001–$24,000	381 (18.6)	8,206 (15.3)
$24,001–$32,000	193 (10.7)	5,203 (9.6)
$32,001–$48,000	205 (12.2)	5,994 (11.3)
$48,001–$60,000	152 (8.2)	4,500 (8.7)
$60,001 and greater	309 (17.7)	15,465 (34.2)
Marital status		
Unmarried	1,219 (54.3)	24,310 (36.8)
Married	1,019 (45.7)	37,817 (63.2)
Prenatal health insurance		
Private	555 (28.9)	23,770 (44.0)
Medicaid	1,206 (52.9)	24,475 (36.2)
Other^d^	275 (13.7)	3,145 (5.6)
Self-pay or no prenatal care	105 (4.5)	8,174 (14.1)
Parity		
0	751 (35.1)	24,347 (38.8)
1	697 (33.1)	19,497 (33.0)
2	424 (17.4)	10,268 (16.5)
3 to 5	321 (13.1)	7,246 (10.7)
6 or more	40 (1.3)	682 (1.0)
Currently breastfeeding/pumping		
No	1,059 (57.6)	22,809 (39.8)
Yes	814 (42.4)	32,294 (60.2)
Pregnancy intention		
Intended	622 (30.0)	26,778 (45.3)
Mistimed	716 (33.0)	20,861 (34.0)
Unsure/unintended	872 (37.0)	13,536 (20.7)
IPV during pregnancy		
No	1,982 (88.2)	60,346 (98.0)
Yes	239 (11.8)	1,410 (2.0)
WIC during pregnancy		
No	1,018 (49.1)	36,692 (63.1)
Yes	1,190 (50.9)	24,585 (36.9)
Depression during pregnancy^e^		
No	896 (37.8)	54,234 (89.7)
Yes	1,346 (62.2)	7,110 (10.3)

^a^ Information on annual household income and breastfeeding was missing in ∼17% and 12% of participants, respectively.

^b^ Other includes American Indian/Alaska Native, Asian, Native Hawaiian, mixed, and others.

^c^ Annual total household income before taxes during the 12 months before birth.

^d^ Other includes Health Insurance Marketplace and other state-specific options.

^e^ Self-reported having a clinical visit for depression during their most recent pregnancy.

IPV, intimate partner violence; PDS, postpartum depressive symptoms; WIC, Women, Infants, and Children.

Without adjusting for confounding variables, teen moms were significantly more likely to report PDS compared with their older counterparts (OR = 1.82; 95% confidence interval [CI], 1.35–2.47) (Table 2). A positive dose–response relationship was observed between annual household income during the 12 months that preceded delivery and PDS, as the odds of mothers reporting PDS increased as income decreased. Mothers who lived at or below 100% of the FPG during the year before delivery were significantly more likely to experience PDS (OR = 1.66; 95% CI, 1.26–2.18) compared with their maternal counterparts who reported an income of between $48,000 and $60,000 (reference) (Table 2). In addition, unmarried mothers were significantly more likely to experience PDS (OR = 2.04; 95% CI, 1.78–2.22) compared with their married counterparts (reference) (Table 2).

**Table 2.** Unadjusted Odds of Reporting Symptoms of Postpartum Depression Among Pregnancy Risk Assessment Monitoring System Participants, 2016 to 2017 (*N* = 64,421)

Characteristics	uOR	95% CI limits
Maternal age, years		
19 and younger	1.82	1.35–2.47
20–29	1.67	1.44–1.93
30–39	1.00	Reference
40 and older	0.75	0.48–1.15
Race		
White	1.00	Reference
Black	1.02	0.85–1.22
Other	0.77	0.63–0.93
Ethnicity		
Non-Hispanic	1.00	Reference
Hispanic	0.72	0.59–0.89
Education		
Less than high school	0.72	0.58–0.90
High school graduate/GED	1.00	Reference
Greater than high school	0.65	0.56–0.76
Annual household income		
$0 - $16,000	1.66	1.26–2.18
$16,001–$24,000	1.29	0.96–1.74
$24,001–$32,000	1.18	0.84–1.66
$32,001–$48,000	1.15	0.83–1.58
$48,001–$60,000	1.00	Reference
$60,001 and greater	0.55	0.41–0.74
Marital status		
Unmarried	2.04	1.78–2.33
Married	1.00	Reference
Prenatal health insurance		
Private	1.00	Reference
Medicaid	2.22	1.90–2.60
Other	1.47	1.16–1.87
Self-pay or no prenatal care	1.23	0.87–1.73
Parity		
0	0.90	0.76–1.07
1	1.00	Reference
2	1.05	0.87–1.28
3 to 5	1.22	0.99–1.51
6 or more	1.29	0.77–2.17
Currently breastfeeding/pumping		
No	2.06	1.77–2.38
Yes	1.00	Reference
Pregnancy intention		
Intended	1.00	Reference
Mistimed	1.47	1.23–1.75
Unsure/unintended	2.70	2.28–3.19
IPV during pregnancy		
No	1.00	Reference
Yes	6.44	5.08–8.17
WIC during pregnancy		
No	1.00	Reference
Yes	1.78	1.55–2.03
Depression during pregnancy		
No	1.00	Reference
Yes	14.33	12.43–16.52

CI, confidence interval; IPV, intimate partner violence; uOR, unadjusted odds ratios.

Similarly, mothers who were not breastfeeding/pumping at the time of survey completion were approximately two times (OR = 2.06; 95% CI, 1.77–2.38) as likely as mothers who were breastfeeding/pumping (reference) to experience PDS (Table 2). Furthermore, mothers who reported their most recent pregnancy to be mistimed (*i.e.*, too soon or too late) or unintended were significantly more likely to experience PDS (OR = 1.47; 95% CI, 1.23–1.75) (OR = 2.70; 95% CI, 2.28–3.19), respectively, compared with mothers who reported their pregnancy to be both intended and well-timed (Table 2). The odds of reporting PDS among mothers who experienced IPV during pregnancy were six times (OR = 6.44; 95% CI, 5.08–8.17) as likely as their maternal counterparts who did not experience violence during pregnancy (reference) to report experiencing depressive symptoms (Table 2).

Of the 2,217 mothers who self-reported having a clinical visit for depression during their most recent pregnancy, 60.7% experienced PDS. After stratifying by depression status during pregnancy and controlling for the confounding effects of maternal age, race, and ethnicity, aORs for the associations between select social determinants and PDS are presented in Table 3. Mothers ages 20–29 years who did not experience depression during pregnancy were most likely to report experiencing PDS (OR = 1.52; 95% CI, 1.14–2.02) (Table 3). Among mothers who did not have antenatal depression, those of Hispanic ethnicity were less likely to report experiencing PDS compared with the non-Hispanic referent group (OR = 0.72; 95% CI, 0.47–1.09) (Table 3). The dose–response effect between income and PDS was no longer observable.

**Table 3.** Adjusted Odds of Reporting Symptoms of Postpartum Depression Among Pregnancy Risk Assessment Monitoring System Participants With and Without a Clinical History of Depression During Pregnancy, 2016 to 2017 (*N* = 2,242)

	PDS among those with depression during pregnancy(***n*** = 1,346)		PDS among those without depression during pregnancy(***n*** = 896)	
	**aOR**	**95% CI limits**	**aOR**	**95% CI limits**
Maternal age, years				
19 and younger	0.87	0.52–1.47	1.22	0.64–2.32
20–29	1.22	0.93–1.60	1.52	1.14–2.02
30–39	1.00	Reference	1.00	Reference
40 and older	1.05	0.51–2.16	0.76	0.34–1.69
Race				
White	1.00	Reference	1.00	Reference
Black	0.78	0.56–1.08	0.75	0.51–1.11
Other	0.82	0.58–1.17	1.08	0.77–1.51
Ethnicity				
Non-Hispanic	1.00	Reference	1.00	Reference
Hispanic	0.87	0.61–1.24	0.72	0.47–1.09
Annual household income				
$0–$16,000	0.67	0.43–1.04	1.21	0.72–2.04
$16,001–$24,000	0.66	0.42–1.04	1.20	0.68–2.11
$24,001–$32,000	0.65	0.37–1.11	1.27	0.73–2.23
$32,001–$48,000	0.80	0.49–1.30	1.54	0.91–2.62
$48,001–$60,000	1.00	Reference	1.00	Reference
$60,001 and greater	0.74	0.47–1.17	1.14	0.70–1.85
Marital status				
Unmarried	1.22	0.91–1.62	1.03	0.71–1.48
Married	1.00	Reference	1.00	Reference
Currently breastfeeding/pumping				
No	1.41	1.09–1.82	1.27	0.95–1.71
Yes	1.00	Reference	1.00	Reference
Pregnancy intention				
Intended	1.00	Reference	1.00	Reference
Mistimed	1.16	0.87–1.53	0.99	0.73–1.35
Unsure/unintended	1.36	1.01–1.82	1.65	1.19–2.27
IPV during pregnancy				
No	1.00	Reference	1.00	Reference
Yes	2.44	1.68–3.55	3.60	2.12–6.12

aOR, adjusted odds ratios; adjusted for maternal age, race, ethnicity.

Irrespective of depression status during pregnancy, unmarried mothers were slightly more likely to report PDS than their married counterparts; however, this result was nonsignificant (Table 3). Similarly, mothers who were not breastfeeding remained more likely to report PDS. The odds of reporting PDS among mothers with antenatal depression who were not breastfeeding were significantly greater than their breastfeeding counterparts (OR = 1.41; 95% CI, 1.09–1.82). Factors such as pregnancy intendedness and intimate partner violence remained significant predictors for experiencing PDS, irrespective of the mothers' antenatal depression status. Mothers who considered their pregnancy to be unintended or were unsure about the intendedness were significantly more likely (OR = 1.36; 95% CI, 1.01–1.82), (OR = 1.65; 1.19–2.27) to report PDS, respectively, when compared with their maternal counterparts with intended, well-timed pregnancies (reference) (Table 3).

Among mothers who were depressed and experienced IPV during pregnancy, the odds of experiencing PDS were nearly 2.5 times (OR = 2.44; 95% CI, 1.68–3.55) as likely as those of mothers who were depressed during pregnancy and had not experienced IPV (Table 3). The odds of experiencing PDS among mothers who were not depressed but had experienced IPV during pregnancy were nearly four times (OR = 3.60; 95% CI, 2.12–6.12) as likely as those of mothers who were both not depressed and had not experienced IPV during pregnancy (Table 3).

## Discussion

In a review of relevant literature, biological or psychological risk factors for PPD have been well-established, while research exploring relationships between SDOH and maternal mental health continues to grow.^9,10^ This study presents insight into the prevalence of mothers experiencing PDS and the social contexts from which this outcome derives.

Studies conducted in 2001 and 2004 by Beck and Robertson et al., respectively, demonstrated that depression before and during pregnancy to be a significant risk factor for developing PPD.^42,43^ The results of our study are in agreement with such findings, as mothers with a history of antenatal depression were significantly more likely to experience symptoms of PDS when compared with mothers without a history of depression during pregnancy. In addition, our findings were also in agreement with those of Beck, who found thirteen predictors of PPD, including unintended pregnancy, social network support, and psychosocial stress.^42^ Lastly, the findings of our study lend support to the results of a 2012 meta-analysis completed by Beydoun et al. that demonstrated a twofold increased risk of symptoms of PDS and PPD among women who experienced IPV compared with women who did not experience IPV.^37^

The PRAMS data set adjusts for underrepresentation and overrepresentation, which increases the discernibility of results to the U.S. population of new mothers. The findings in this study are subject to several limitations. First, PRAMS is a cross-sectional questionnaire; thus, causality cannot be established. Results only represent women with a recent live birth in the PRAMS sites* included in the report. Women who reside in any site that did not meet the response threshold were excluded, as well as women with a recent miscarriage, stillbirth, or spontaneous abortion. Symptoms of PPD were self-reported and are not indicative of a clinical diagnosis of depression. Results may not capture symptoms that resolved before or began after survey completion.

Furthermore, the complexities of a mother's social network were challenging to measure using the core questionnaire and birth certificate data. Since this study's data were collected, the CDC developed and implemented a PRAMS module that includes measures to assess housing and food security, education, financial stability, and health care access.^44^ Lastly, this study did not measure all factors associated with experiencing PDS. Factors such as complications during pregnancy or delivery and birth outcomes should be considered in future research. Despite these limitations, PRAMS continues to be an invaluable source of maternal and child health data due to its standardized collection methodology. Only data from states that have met the relatively high response rate threshold are included in the sample, representing ∼83% of all live births in the United States. The potential for reporting bias is minimal due to PRAMS survey responses being anonymous.

Our results further demonstrate the importance of public policies (*e.g.*, legislation that mandates education on and screening for PDS) and multifaceted social programs that assist new mothers during the prenatal and postpartum periods.^45^ An example of a multifaceted social program is the WIC program, which integrates a PPD screening into their everyday practice while collaborating with community-based behavioral health services to provide referrals and treatment.^41^ The 2022 White House Blueprint for Addressing the Maternal Health Crisis includes expanding the National Maternal Mental Health Hotline for new mothers to increase access to behavioral health care.^16^ The Blueprint also called for expanding the capability to screen, treat, and refer for PPD and integrating behavioral health supports in community settings (*e.g.*, school-based health centers and community-based organizations).^16^

Employing the power of health policy and programs to increase opportunities for maternal mental health screenings would be ineffective without addressing the stigmatization new mothers feel when they speak honestly about their emotional journeys during the postpartum period. To reduce this societal stigma, the 2022 White House Blueprint for Addressing the Maternal Health Crisis included a plan for the U.S. Department of Health and Human Services to facilitate a media campaign to increase knowledge and raise awareness for women, families, and the public about PPD.^16^

## Conclusion

In the last decade, there has been an increasing awareness of the importance of addressing SDOH as a comprehensive strategy for improving health, particularly among groups disproportionally affected by SDOH. While access to quality health care is essential, social risk factors are estimated to drive as much as half of health outcomes. This study demonstrates that in addition to a history of antenatal depression, several SDOH significantly impact the likelihood of experiencing depressive symptoms postpartum. The likelihood of experiencing PDS is particularly significant among lower socioeconomic status mothers, especially those with inadequate social network support.

## Data Availability

Data analyzed in this study were a secondary analysis of existing data, which are openly available at locations cited in the Reference section.^13^ Further documentation about data processing is available at the CDC's PRAMS website at www.cdc.gov/prams/prams-data/researchers.htm

## Abbreviations Used

aOR: adjusted odds ratio
CDC: Centers for Disease Control and Prevention
CI: confidence interval
FPG: Federal Poverty Guidelines
IPV: intimate partner violence
ORs: odds ratios
PDS: postpartum depressive symptoms
PPD: postpartum depression
PRAMS: Pregnancy Risk Assessment Monitoring System
SDOH: social determinants of health

## Notes

^*^
The 30 sites included Alaska, Arkansas, Colorado, Connecticut, Delaware, Illinois, Iowa, Louisiana, Maine, Maryland, Massachusetts, Michigan, Missouri, Nebraska, New Hampshire, New Jersey, New Mexico, New York, New York City, Oklahoma, Pennsylvania, Rhode Island, Utah, Vermont, Virginia, Washington, West Virginia, Wisconsin, and Wyoming.
